# Neuroimmunological investigations of cerebrospinal fluid in patients with recent onset depression – a study protocol

**DOI:** 10.1186/s12888-021-03633-0

**Published:** 2022-01-12

**Authors:** Nina Vindegaard Sørensen, Sonja Orlovska-Waast, Rose Jeppesen, Rune Haubo Christensen, Michael Eriksen Benros

**Affiliations:** 1 Biological and Precision Psychiatry, Copenhagen Research Centre for Mental Health, Mental Health Centre Copenhagen, Copenhagen University Hospital, Gentofte Hospitalsvej 15, 4. sal, 2900 Hellerup, Denmark; 2 Department of Immunology and Microbiology, Faculty of Health and Medical Sciences, University of Copenhagen, Copenhagen, Denmark

**Keywords:** Depression, Immunology, Cerebrospinal fluid, Biomarkers, Cytokines

## Abstract

**Background:**

A proinflammatory response has been suggested to be involved in the pathophysiology of depression in a subgroup of patients. However, comprehensive largescale studies on neuroimmunological investigations of the cerebrospinal fluid (CSF) are lacking and no largescale longitudinal CSF studies comparing patients with depression to healthy controls currently exist.

**Methods:**

A longitudinal case-control study including at least 100 patients with first time depression (ICD-10: F32) within the past year with ongoing symptoms and at least 100 sex and age matched healthy controls with collection of CSF, blood, and fecal samples. All individuals will be evaluated by neurological examination including neurological soft signs, interviewed for psychopathology assessment and have symptomatology evaluated by relevant rating scales. Level of functioning and quality of life will be evaluated by a panel of interview questions and rating scales, and cognitive function assessed by a relevant test battery. In addition, a large number of potential confounders will be registered (BMI, smoking status, current medication etc.). *Primary outcomes:* CSF white cell count, CSF/serum albumin ratio, CSF total protein levels, IgG index, CSF levels of IL-6 and IL-8, and the prevalence of any CNS-reactive autoantibody in CSF and/or blood. *Secondary outcomes:* exploratory analyses of a wide range of neuroimmunological markers and specific autoantibodies. Power calculations are computed for all primary outcomes based on previous CSF studies including patients with depression and healthy controls.

**Discussion:**

This study will represent the hitherto largest investigation of CSF in patients with recent onset depression compared to healthy controls. We expect to elucidate neuroimmunological alterations in individuals with depression and characterize an immunological profile paving the way for the development of effective treatments based on biomarkers.

**Trial registration:**

The study is approved by The Regional Committee on Health Research Ethics (Capital Region, j.no: H-16030985) and The Danish Data Protection Agency (j.no: RHP-2016-020, I-Suite no.: 04945).

**Supplementary Information:**

The online version contains supplementary material available at 10.1186/s12888-021-03633-0.

## Background

The underlying pathophysiology of depression is heterogeneous and yet not fully understood. In this study we hypothesize that the pathophysiology in a subgroup of patients is caused by altered immunological activation and/or function, as it has been suggested by several authors [1–3]. This hypothesis is based on a magnitude of associations and findings from many diverse studies. Epidemiological studies have revealed that immune-related diseases, such as severe infections and autoimmune disorders, are associated with an increased risk of depression [4, 5], while a genome-wide meta-analysis of depression studies has revealed 102 independent gene variants related to depression including genes related to the immune system [6]. A recent meta-analysis investigating immunological differences in blood samples from patients with depression compared to controls have amongst others found interleukin-6 (IL-6) and IL-8 to be elevated in patients with depression [7]. The most pronounced inflammatory response in blood is seen in the acute phase of illness, and a decrease in e.g. IL-6 levels is observed following treatment [8]. Blood C-reactive protein (CRP) levels have additionally been associated with symptom severity in women [9], and meta-analyses of placebo controlled randomized controlled trials (RCTs) with anti-inflammatory treatment have shown beneficial effects on depression and depressive symptoms [10, 11]. Together, these findings indicate that a proinflammatory or dysfunctional immunological activation can contribute to the development of depression in a subgroup of patients.

However, the blood is separated from the brain by the blood-brain-barrier and the blood-cerebrospinal fluid (CSF)-barrier - that we hereafter refer to commonly as the blood-brain-barrier (BBB) as this expression is more commonly used in the literature. Measuring of immunological markers in the CSF, the material closest to the brain obtainable from living patients, is the gold standard when conducting studies of neuroinflammation. White cell count (WCC) in CSF is one of the most direct measures of increased immune cell activity within the brain, but is an unspecific indicator of pathological changes, since it can be caused by a variety of disorders [12]. Cytokines are also markers of immune cell activity and a meta-analysis of CSF samples comparing patients with depression to controls found increased levels of the pro-inflammatory cytokines IL-6 and IL-8 in CSF [13]. These cytokines are produced by a variety of cells and are of particular interest when investigating the pathophysiology of depression due to their pro-inflammatory properties [14]. Another marker of increased inflammatory activity is an impaired BBB, since inflammation can increase BBB permeability [15]. A meta-analysis of patients with affective disorders compared to healthy controls showed increased levels of CSF/serum albumin ratio and CSF total protein in patients [16], indicating a more permeable BBB [17, 18] among patients. The most reliable marker of BBB impairment is the CSF/serum albumin ratio [17], but studies on CSF from patients with depression compared to healthy controls are few and small with conflicting results [19, 20]. Intrathecal synthesis of IgG is observed in a variety of inflammatory diseases [21], some of them, e.g. multiple sclerosis [22], with substantial symptom overlap/co-morbidity with depression, but one of the most reliable markers of intrathecal IgG synthesis, the IgG index [21], is only investigated in one small previous study [19]. Autoimmune encephalitis [23] can also cause depression or depressive symptoms; however, no previous studies have compared CNS-reactive autoantibodies in CSF between patients with depression and healthy controls [24]. Furthermore, as a relatively recent hypothesis, the gut microbiota – all the microbes in the gut – has been suggested as a potential contributor to depression pathophysiology [25] mediated by the immune system [26]. The largest study until date included 156 patients with depression and 155 healthy controls and revealed disturbances of the gut microbiota to be related to depression [27]; however, large longitudinal studies are warranted [25] and investigations related to CSF biomarkers of immune system alterations are missing.

Overall previous CSF biomarker studies of patients with depression compared to healthy controls vary in sample size with markers like IL-8 and CSF total protein measured in large cohorts of patients (*n* = 104 [28] and *n* = 90 [29], respectively), while other important markers like IL-6 and IgG index are measured only in rather small cohorts of patients (*n* = 30 [30] and *n* = 29 [19], respectively). Most of the current CSF studies on inflammatory biomarkers in depression do not have healthy comparison groups, making the interpretation of the results difficult, and no largescale longitudinal studies have investigated neuroimmunological markers related to depression.

In the present study, the most promising biomarkers of neuroinflammation related to depression will be investigated including total CSF WCC, CSF/serum albumin ratio, CSF total protein, IgG index, CSF levels of IL-6 and IL-8, and any CNS-reactive autoantibody in CSF and/or blood. Additionally, exploratory analyses of a broad panel of neuroimmunological markers and specific analyses of CNS-reactive autoantibodies will be carried out. This will be the hitherto most extensive investigation of immune-related changes in CSF, blood and gut microbiota from a large cohort of patients with a recent onset first time depression diagnosis compared to healthy controls and the first study to assess this longitudinally, hereby paving the way for a more precise understanding of the pathophysiology and treatment of depression.

## Methods/design

**Fig. 1:**
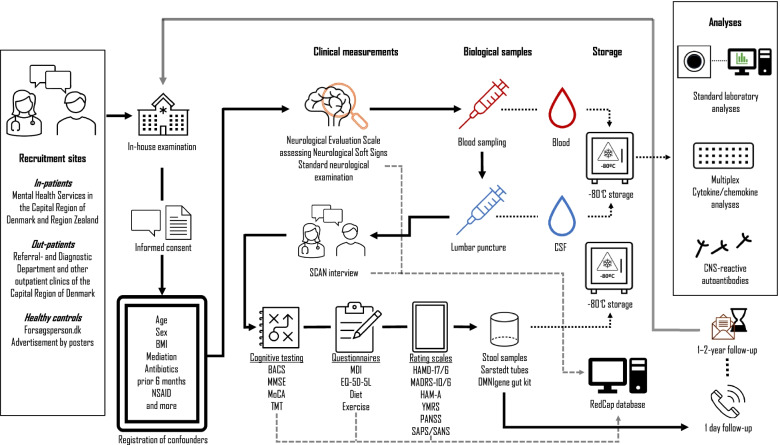
Recruitment, inclusion and follow-up. Patients will mainly be recruited from the Referral and Diagnostic Department and healthy volunteers from internet advertisement. Before any examination, the participant will sign the informed consent formula. A variety of potential confounders will be registered by questioning or questionnaires (including body-mass index (BMI), smoking status, current psychotropic and other medication, non-steroid anti-inflammatory drugs (NSAID), alcohol, eating and exercise habits and more). Contraindications to lumbar puncture will be evaluated. Prior to lumbar puncture a neurological examination including neurological evaluation scale to assess neurological soft signs will be performed and blood samples drawn. After the lumbar puncture the WHO Schedules for Clinical Assessment in Neuropsychiatry (SCAN) interview will be conducted (SCAN interview will be conducted prior to lumbar puncture for healthy participants to rule out prior and current psychiatric symptoms). Cognitive testing includes Brief Assessment of Cognition in Schizophrenia (BACS), Montreal Cognitive Assessment (MoCA), Mini-Mental State Examination (MMSE) and Trail Making Test (TMT) A and B. Questionnaires with self-rating of depressive symptoms (Major Depression Inventory (MDI)), quality of life (EQ-5D-5L), diet and exercise will be filled in by the participant. After the visit the following rating scales will be evaluated by the research assistant: Symptom rating (depressive symptoms: Hamilton depression rating scale – 17 items (HAMD-17)/−6 items (HAMD-6) and Montgomery-Asberg Depression Rating Scale - 10 items (MADRS-10)/− 6 items (MADRS-6), manic symptoms: Young Mania Rating Scale (YMRS), anxiety symptoms: Hamilton Anxiety Rating Scale (HAM-A) and psychotic symptoms: Positive and Negative Symptom Scale (PANSS) and Scale for Assessment of Positive/Negative Symptoms (SAPS/SANS)) and functioning by Personal and Social Performance Scale (PSP) and Global Assessment of Functioning. Fecal samples will be collected at home. The participant will be followed up the day after the intervention for the registration of possible side effects. All biological samples will be stored in a biobank for later laboratory analyses. The participant will be followed up after one-two years with repetition of all measurements

An overview of the study process is provided in Fig. 1.

### Study design

A prospective case-control study of a minimum of 100 patients with first time depression (International Classification of Diseases 10th Revision (ICD-10): F32) and a minimum of 100 age and sex matched healthy controls.

### Study aims

The aim of this study is to identify neuroinflammatory alterations that contribute to the development of depression by longitudinal examinations of CSF, blood and gut microbiota together with thorough psychopathological and neurological examination leading to a more detailed understanding of the interplay between the immune system and the brain.

#### Primary outcomes

The publication plan with elaboration on primary outcomes is given in Table 1. In the neuroinflammation study the four co-primary outcomes are CSF total WCC, CSF/serum albumin ratio, CSF total protein, and IgG index. In the cytokine study the two co-primary outcomes are CSF levels of IL-6 and IL-8, and in the CNS-reactive autoantibody study the two co-primary outcomes are 1) the presence of any one of seven (see Table 1) CNS-reactive autoantibodies in CSF and 2) CNS-reactive autoantibodies in CSF or blood.

**Table 1:** Publication plan

Publication	Outcomes		Elaboration
*Neuroinflammation study*	Primary	Cerebrospinal fluid (CSF) total white cell count (WCC), CSF/serum albumin ratio, CSF total protein, and immunoglobulin G (IgG) index	CSF total WCC is one of the most valuable CSF markers in regards of inflammation [31], but to our knowledge not yet investigated by more precise methods than standard analysis in the context of depression. Standard analysis has the disadvantage that a cell count < 3-5 will not usually be reported leading to interpretation difficulties of small differences in WCC. However, such differences could very well be of importance when investigating low grade inflammation. Increased blood-brain-barrier (BBB) permeability can be related to neuroinflammation [15], and the best marker hereof is CSF/serum albumin ratio [32]. CSF total protein is also increased due to BBB impairment, but is, less specifically however, increased in a broad variety of neurological diseases [33]. IgG index is often used to screen for inflammatory central nervous system (CNS) diseases and reflects intrathecal IgG production [21].
	Secondary	CSF WCC differential count, CSF neutrophil/lymphocyte ratio, CSF/serum IgG ratio, and CSF/plasma glucose ratio	Depending on the degree and predominant cell type, CSF pleocytosis is seen in a variety of brain disorders, both infectious and non-infectious [12]. The ratio between neutrophils and lymphocytes is a marker of inflammation and has been found elevated in blood among patients with depression compared to healthy controls [34]. Increased CSF/serum IgG might reflect increased IgG production within tectum [16]. Severe CNS infections can decrease CSF/plasma glucose ratio [12].
*CSF cytokine and chemokine profile study*	Primary	CSF interleukin (IL-6) and interleukin-8 (IL-8)	The innate immune system is hypothesized to play an important role in the pathophysiology of depression with IL-6 as an important marker of this type of inflammation [3], while IL-8 is mainly involved in diapedeses and chemotaxis of leukocytes [35]. A recent meta-analysis revealed a significant increase in IL-6 and IL-8 in CSF from patients with depression compared to controls, but also revealed how most previous studies had small sample sizes and conflicting results [13].
	Secondary	ICAM-1, IFN-γ, IL-1α, IL-1β, IL-2, IL-4, IL-5, IL-7, IL-10, IL-12/IL-23p40, IL-13, IL-15, IL-16, IL-17A, IP-10, MCP-1, MCP-4, MDC, MIP-1α, MIP-1β, TARC, TNF-α, TNF-β	To exploratively investigate contrasts between groups, a broad cytokine panel analysis will be carried out.
*CNS-reactive autoantibody study*	Primary	Any CSF CNS-reactive antibody of either glutamate receptor (type NMDA), glutamate receptor (type AMPA1), glutamate receptor (type AMPA2), CASPR2, LGI1, GABA B receptor or GAD65 in 1) CSF or 2) CSF or blood.	There is a well-established association between autoimmune disorders and depression [4] and also an overlap between the symptomatology of autoimmune and depressive disorders [23]. No previous studies have investigated CNS-reactive autoantibodies in CSF from patients with depression compared to healthy controls [24].
	Secondary	Specific CSF CNS-reactive antibodies in CSF and/or blood.	The specific prevalence of the above-mentioned CNS-reactive autoantibodies in CSF and/or blood will provide a thorough characterization of the humoral response towards CNS.

Abbreviations: *CSF* cerebrospinal fluid, *IgG* immunoglobulin G, *WCC* white cell count, *IL* interleukin, *ICAM* intercellular adhesion molecule 1, *IFN* interferon, *MCP* monocyte chemoattractant protein, *MDC* macrophage derived protein, *TARC* thymus- and activation-regulated chemokine, *TNF* tumor necrosis factor, *CNS* central nervous system, *NMDA* N-methyl-d-aspartate, *AMPA* alpha-amino-3-hydroxy-5-methyl-4-isoxazolepropionic acid, *CASPR2* contactin-associated protein 2, *LGI1* leucine-rich glioma-inactivated protein 1, *GABA* gamma-Aminobutyric acid, *GAD65* glutamic acid decarboxylase-65

#### Secondary outcomes

To exploratively search for differences between patients and healthy controls in a broad variety of neuroimmunological markers, CSF cytokines and chemokines, and specific analyses of CNS-reactive autoantibodies in CSF and/or blood. The secondary outcomes are specified in Table 1.

### Setting of study

The study intervention will be conducted at the facility of Biological and Precision Psychiatry, Copenhagen Research Centre for Mental Health (CORE), Mental Health Centre Copenhagen, Copenhagen, Denmark or where the patient is hospitalized. The study intervention will always take place in an undisturbed room. Patients will be offered transport if needed.

### Participant eligibility criteria

#### Inclusion criteria for cases

1. Patients with a first-time diagnosis of a depressive disorder (according to ICD-10: F32) diagnosed within the past year.
2. Ongoing depressive symptoms.
3. Age between 18 and 50 years*.*
4. Obtainment of written informed consent.

#### Inclusion criteria for healthy controls

1. Healthy individual.
2. Age between 18 and 50 years.
3. Obtainment of written informed consent.

The healthy controls will preferably be matched with patients in accordance to sex and age.

The exclusion criteria are described and explained in Table 2.

**Table 2:** Exclusion criteria

Exclusion criteria	Explanation
*All participants*	
**1.** Prior diagnosis within ICD-10 F20-39	All potential patients are screened for and excluded in the case of a history of psychotic or affective disorders, since we aim to include patients with first-time depression only.
**2.** Contraindications against lumbar puncture	To minimize the risk of serious side effects potential participants with contraindication against lumbar puncture will not be included. Contraindications includes increased risk of bleeding (known International Normalized Ratio (INR) > 1.5, blood platelets < 40 × 10^9^/L, blood thinning treatment), signs of increased intracranial pressure (postural headache, recent onset morning headache, nausea) [36] or fever.
**3.** Known organic psychiatric disorder or severe neurological disorder	To minimize the impact of other sources of neuroinflammation, patients with known organic cause to their symptoms (e.g. encephalitis), and/or known organic psychiatric disorder (ICD-10 F0), and/or known severe neurological disorder(s), including brain tumor, stroke, multiple sclerosis, epilepsy (with seizures within the past 10 years), and/or severe head injury within the past 3 months are not considered eligible.
**4.** Severe somatic disease	To reduce the impact of other sources of inflammation, participants with diseases known to have major impact on the immune system (including active infection, cancer, autoimmune disorders (e.g. inflammatory bowel disease, or systemic lupus erythematosus), hypothyroidism or hyperthyroidism) are not considered eligible. However, participants with mild asthmatic bronchitis, allergy, or other common, mild somatic disorders will be included in both groups in order to avoid selection bias.
**5.** Regular use of anti-inflammatory medication	To reduce the impact of medication impacting the immune system all participants are screened for the use of anti-inflammatory or immunosuppressive drugs (including Non-steroidal Anti-inflammatory Drugs, cortisone treatment (orally or intravenous), monoclonal antibody therapy of any kind and plasmapheresis). The participant can be included after a quarantine period of 14 days if still fulfilling the remaining eligibility criteria.
**6.** Current electroshock therapy (ECT)	ECT is known to induce a proinflammatory response [37] and current ECT will lead to exclusion to avoid interference with results. After a 3-month quarantine the participant can be included if still fulfilling the inclusion criteria.
**7.** Current abuse of alcohol or drugs	Potential participants who full-fill the criteria of a F1 diagnosis will not be included to avoid interference with results, however, recreational use will be accepted, also among healthy participants, since we aim for highly translational results. If the use of alcohol or drugs could have caused the current psychiatric illness or if the use is on regular (daily) basis, the participant will not be included.
**8.** Not being able to participate in SCAN interview	Patients with minor psychiatric co-morbidity (e.g. anxiety) can be included, but if the participant has psychiatric co-morbidity complicating SCAN interview (e.g. severe autism or mental retardation), they will not be included. Participants who cannot participate in a (full) SCAN interview due to their depressive state will still be included.
**9.** Language barrier	We aim for a thorough characterization of psychopathology in this study, and it will be individually evaluated whether a participant should be excluded due to language barrier, however, the participant should be capable of answering the questions of the SCAN interview. The interview will be conducted in either Danish or English.
**10.** Pregnancy	As a precautionary principle, pregnant women are not allowed to participate. Furthermore, pregnancy is known to impact the female body in multiple ways including alterations of the immune system [38]. Female participants are asked routinely about their menstrual cycle and hormonal contraception, and a risk of pregnancy or known pregnancy leads to exclusion from the study. Lactating women can participate.
**11.** Any psychiatric disorder (or former psychiatric disorder)	All healthy controls are screened for prior psychiatric disorders. Initially by phone, but also during the SCAN interview carried out before blood samples and lumbar puncture. The SCAN interview is based on the past 28 days, but all participants will be asked if they ever experienced any of the symptoms, and if the interview reveals possible former psychiatric disorder the healthy control is excluded. Furthermore, individuals treated with psychotropic medication for any reason will be excluded from the healthy control group.

### Sample recruitment

Patients are recruited from the in- and outpatient facilities at The Mental Health Services of the Capital Region of Denmark, and a majority of the patients will be recruited from the Referral- and Diagnostic Department (RDD). For a more detailed description see eSupplementary paragraph 1.

Healthy controls will mainly be recruited via a Danish web portal used to find participants for clinical studies (www.forsøgsperson.dk) and will thus represent community controls originating from the same geographic area as the patients in accordance to the Newcastle-Ottawa quality assessment scale for case control studies (NOS) [39].

### Study procedure

#### Information and screening assessment

All participants are screened according to the eligibility criteria prior to the date of enrolment. They are all informed both orally and in writing prior to the enrolment with sufficient time to consider participation. For a detailed description of this procedure see eSupplementary paragraph 2.

#### Sampling and data recording

For each participant, we aim to perform all clinical measurements and biological samples on the same day. The inclusion starts approximately at 9:00 a.m., but due to feasibility the starting time can fluctuate between 8:00-10:00 a.m. The study program is scheduled to last 3-5 h and will vary depending on symptom severity and the participant’s need for breaks. For a detailed description of the study time schedule see eSupplementary Paragraph 3 and eTable 1.

#### Consent

Before any examination is carried out, informed written consent for participation is obtained (for more details, see eSupplementary Paragraph 2).

#### Clinical measures

All participants will undergo an interview using WHO Schedules for Clinical Assessment in Neuropsychiatry (SCAN) [40], including chapters 4 (anxiety symptoms), 6, 7, 8 (depressive symptoms), 10 (manic symptoms), 16, 17, 18 and 19 (psychotic symptoms), since these chapters cover the most severe psychiatric symptoms with the highest relevance to our study. The purposes of the SCAN-interview will be two: 1) for patients to confirm the depression diagnosis given by the clinician and to characterize symptomatology, 2) for healthy controls to exclude present or prior psychiatric illness. All interviewers are certified in performing SCAN interviews. Healthy controls will be interviewed prior to blood sampling and lumbar puncture, to ensure that no psychiatric disorders are present. All participants will undergo a thorough neurological examination including Neurological Evaluation Scale (NES) for evaluation of Neurological Soft Signs (NSS) as described by Dazzan et al. [41] prior to lumbar puncture.

Some questions have been added to the interview of the participants in order for the interviewers to afterwards be able to rate the participant on the following psychopathology rating scales; 17-item Hamilton Depression Rating Scale (HAMD-17) [42], 6-item Hamilton Depression Rating Scale (HAMD-6) [43], 10-item Montgomery-Asberg Depression Rating Scale (MADRS-10) [44] and 6-item Montgomery-Asberg Depression Rating Scale (MADRS-6) [45], Hamilton Anxiety Rating Scale (HAM-A) [46], Positive and Negative Symptom Scale (PANSS) [47], Scale for Assessment of Positive/Negative Symptoms (SAPS/SANS) [48], and Young Mania Rating Scale (YMRS) [49]. Functioning will be assessed using Personal and Social Performance Scale (PSP) [50] and Global Assessment of Functioning (GAF) [51].

During the visit, all participants will be asked to complete a set of questionnaires including Major Depression Inventory (MDI) [52], and quality of life by EQ-5D-5L and visual analogue scale of Quality of Life (VAS QL) [53].

Cognitive testing will be done using the Brief Assessment of Cognition in Schizophrenia (BACS) [54], Montreal Cognitive Assessment (MoCA) [55], Mini-Mental State Examination (MMSE) [56] and Trail Making Test (TMT) A and B [57]. All testers are certified in using BACS.

#### Other self-reported measures

The following self-reported data is collected; height, weight, smoking status, alcohol intake, use of recreational drugs, allergies, somatic illnesses, prior and concurrent psychiatric disorders, current medication (both psychotropic and non-psychotropic), intake of non-steroid anti-inflammatory drugs, paracetamol and antihistamines in the prior 2 weeks, use of antibiotics in the prior six months, information on participants menstrual cycle, time for last intake of food and years of education. Information on prior and concurrent psychiatric disorders and medication status/duration is confirmed by the medical record as well. Information on current diet and exercise habits will be provided from a modified version of a questionnaire [58] used for evaluation of national health in Denmark by the Danish Health Authority [59] (eSupplementary paragraph 6).

#### Quality control

To minimize interrater variability, we will regularly conduct co-evaluation of patients with main focus on rating scales. Two research assistants will participate in a full participant inclusion and will individually be diagnosing and rating the participant. Any discrepancies will be noted and discussed with a specialist in psychiatry.

#### Validation of diagnosis – case definition

In accordance with the NOS, the gold standard for an adequate case definition is independent validation. All patients will be evaluated by SCAN interview. Additionally, patients who require hospital-based treatment (in- or outpatients) are evaluated by the treating psychiatrist in the clinic before referral to the study. Patients who are not treated at in- or outpatient facilities of the psychiatric hospitals (e.g. followed by a private psychiatrist) will be either independently evaluated by an experienced psychiatrist who will watch the video recording of the SCAN interview or by consensus diagnosis with an experienced specialist in psychiatry. The SCAN interviewer will not be blinded to patient status or diagnosis.

#### Follow-up

The patients will be invited to a follow-up visit after one to two years after enrolment. This follow-up visit is planned to include all the same measurements (including blood and CSF measurements) as for the primary visit and a Questionnaire about the Process of Recovery (QPR) will be added [60]. By the follow-up assessment we expect to collect data that can enhance the understanding of the association between neuroinflammation and depression/depressive symptoms. Furthermore, trajectories of neuroinflammatory markers will be addressed by follow-up of later psychiatric diagnoses in the well validated Danish registers, also for participants who do not attend follow-up visit, in order to search for predictors of disorder progression. We will in detail conduct an analysis plan for the analyses of the follow-up assessments before the end of follow-up inclusion and prior to data analyses.

#### Biological samples and measures

To minimize the impact of diurnal variation all samples are to be collected between 8.30 a.m. and 01.00 p.m.

#### Blood samples

Venous blood samples will be collected prior to lumbar puncture and we aim to have ≥90% of blood samples collected between 9.30 and 11.30 a.m. Prior to the blood sample, a skin swab is performed and stored at −80°C for quality control analyses. A maximum of 44 mL of blood will be sampled; 8 mL used for initial analyses and 36 mL stored for later use. For a detailed description of blood sample drawing and equipment, see eSupplementary Paragraph 4.1.

#### Cerebrospinal fluid samples

The lumbar puncture will be carried out according to current consensus [61], and we aim to have ≥90% of CSF samples collected between 10.00 a.m. and 12.00 p.m. We furthermore aim for the time from collection of blood samples to collection of CSF samples to not exceed 35 min for ≥90% of participants. In brief, the participant will preferably be placed in a lateral decubitus position. The L3/L4 or L4/5 space will be identified, and local anesthesia will be given in the form of a sufficient amount of lidocaine (depending on the size of the participant). Prior to the lumbar puncture, a skin swab will be performed and stored at −80°C for quality control analyses. The lumbar puncture will be carried out preferably using an atraumatic 22G needle. Two mL will be taken for routine examination and 14 mL will be stored in the biobank for later analysis. The samples are to be analyzed as fast as possible and within 1 h from the first droplet of CSF. For details regarding the lumbar puncture, procedure and equipment see eSupplementary Paragraph 4.2.

#### Procedures for biobank storage

Thirty-six mL of blood and 14 mL of CSF will be used for biobank storage and will be stored both on filter papers and portioned in 0.5 ul aliquots after centrifuging at 1145 G for 10 min. For further details of blood and CSF storage procedures, see eSupplementary Paragraph 4.3.

#### Measures of safety regarding lumbar puncture

In order to assess the risk of adverse events associated with lumbar puncture, participants are contacted on the day after the intervention. All symptoms that could be due to the procedure are noted with specific focus on possible post lumbar headache and infections.

#### Fecal samples

Each participant is asked to provide a fecal sample, either during the inclusion, or afterwards from home. Fecal samples are collected in two ways, both in a Sarstedt tube (Sarstedt, Bording, Denmark) and by using the OMNIgene•GUT OMR-200 Sample Collection Kit (DNAgenotek™, Ottawa, Ontario, Canada). If collected at home, the fecal samples will be sent by mail, and then stored in the freezer at − 80 degrees Celsius. Samples done during the visit will be frozen immediately. A score on the Bristol Stool Scale [62], time and date of sampling, as well as time and date of freezing is to be noted. Freeze/thaw cycles are avoided. Since gut microbiota research is fast developing, a detailed analysis plan for the gut microbiota samples is planned to be conducted later on before finishing the longitudinal collection of samples prior to analyses.

#### Other biological measures

Blood pressure and pulse will be measured prior to the neurological examination.

#### Registry data

We will use the Danish registers to obtain information on socioeconomic factors and prior, current and future medical history, such as prior infections treated in the primary care sector, comorbidities, treatment outcome (e.g., psychiatric readmissions and change in medication) and later psychiatric diagnoses.

#### Data storage

All data will be kept in secured folders and in the database RedCap [63, 64] in accordance with approval from The Danish Data Protection Agency. Written consent formulas will be kept in a locked locker in a locked office, and additionally stored electronically in secured folders. For more detailed information on data storage and pseudonymization see eSupplementary Paragraph 4.4.

#### Biological sample storage

All cryo vials will be placed at − 80 degrees Celsius as soon as possible, and time for freezing will be noted. The filter papers will be left to dry overnight at room temperature and placed at − 80 degrees Celsius on the next day. For details see eSupplementary Paragraph 4.3. Fecal samples will be stored at − 80 degrees.

#### Laboratory analyses

All analyses will be run blinded by pseudonymization. Laboratory analyses will be performed according to a statistically designed experimental schedule based on a randomized block design or similar, to minimize the risk of confounding by time of analysis, batch and other factors.

#### Immediate analyses

Eight mL of blood and two mL of CSF will be analyzed immediately at Rigshospitalet. Initial blood analyses include; white blood cell (WBC) count, erythrocytes, hemoglobin, platelets (Sysmex XN9000, Sysmex), high sensitivity CRP, IgG (cobas® 8000 modul c502, Roche), albumin, glucose (cobas® 8000 modul c702, Roche) and HbA1c (Tosoh G8, Sysmex). Initial CSF analyses include; WCC (Sysmex XN9000, Sysmex), differential count (DM96, CellaVision), albumin, IgG, total protein (cobas® 8000 modul c502, Roche), erythrocytes (Sysmex XN9000, Sysmex), glucose and lactate (ABL800 FLEX, Radiometer).

#### Primary outcome analyses

CSF total WCC, CSF/serum albumin ratio, CSF total protein and IgG index will be analyzed immediately as described above. IgG index is calculated by the following formula:

$$\frac{CSF_{IgG}/{Blood}_{IgG}}{CSF_{Albumin}/{Blood}_{Albumin}}$$

CSF Cytokines are planned to be analyzed using the V-plex Neuroinflammation Panel 1 Human Kit from Meso Scale Diagnostics. CSF and blood CNS-reactive autoantibodies are planned to be analyzed by Autoimmune Encephalitis Mosaic 1 and GAD65 panels from Euroimmun.

Primary and secondary outcomes for gut microbiota analysis are yet to be defined due to the fast development of gut microbiota research. However, a detailed analysis plan including well defined primary and secondary outcomes for gut microbiota samples will be conducted prior to finishing the longitudinal collection of samples.

### Statistical analysis plan

Power and sample size calculations are based on two-sample *t*-tests using standardized mean differences (SMDs) and based on not yet published results from a meta-analysis from our group comparing patients with unipolar depression to healthy controls. If all values are observed above the lower limit of quantification (LLOQ), we expect the power for CSF/serum albumin ratio and CSF total protein to be high (95% or higher), whereas the power for IgG index and CSF total WCC are expected to be low (< 50%) though effect sizes in previous studies are heterogenic. For IL-6 we expect good power (> 80%), but low power for IL-8 (< 50%).

Based on previous studies we expect a proportion of biomarker measurements to be lower than LLOQ, most pronounced in the control group. By simulation, we found that given an effect size leading to a power of 80% without censoring, 33% censoring will only reduce power with a maximum of 5%, while 50% censoring will only reduce power with a maximum of 10%, when using the censored Gaussian model (see eSupplementary for further details).

Regarding CNS-reactive autoantibodies the current evidence of expected findings in CSF from healthy controls is very limited. We expect no positive CNS-reactive autoantibodies in the CSF among control subjects [24] and a previous study found no CNS-reactive autoantibodies in CSF from patients either [65]. However, 0.23% of healthy controls [24] and 0.4% of patients with affective syndromes [65] had measurable CNS-reactive autoantibodies in blood.

Linear models will be applied for comparison of all continuous primary outcomes adjusting for sex and age. All continuous outcomes being measured as concentrations or ratios will be log-transformed before analysis (if zeroes are present, a small value, estimated by profile maximum likelihood, will be added to all observations prior to analysis [66]). If values of an outcome are observed below LLOQ, we will use the censored Gaussian model instead of the linear model.

Pearson chi-square test without adjustment for continuity will be applied for analyses of CNS-reactive antibodies. All results of primary and secondary analyses will be reported with the effect size, 95% confidence intervals and *p*-values. Two-sided tests with *p* < 0.05 will be considered significant. All analyses will be done in the program R version 4.0.5 [67] or later using the package “survival” [68] to fit the censored Gaussian model.

## Discussion

### Selection of primary and secondary outcomes

The most recent meta-analyses in this field indicate that large-scale CSF studies of depression are needed [13, 16]. As primary outcomes, total CSF WCC reflects differences in the overall amount of immune cells, while impaired BBB will be explored by CSF/serum albumin ratio and CSF total protein. IL-6 and IL-8 reflecting low grade inflammation are the most relevant cytokines, since activation of the innate immune system in depression pathophysiology seems to be one of the most promising theories [3]. Intrathecal IgG production will be assessed by IgG index and possible autoimmune etiologies will be explored by autoantibody analyses of CSF and blood. These co-primary outcomes have been selected based on the reflections presented in Table 1.

The secondary outcomes will consist of broader and more exploratory analyses of neuroimmunological markers (including CSF differential cell count, lymphocyte/neutrophil ratio, CSF/serum IgG and a broad panel of cytokines and chemokines) and autoantibody profiles hereby providing information for a more thorough biological understanding of the etiology/pathophysiology of depression.

### Selection of study population

We intend to study patients with a first-time diagnosis of depression within the past year, since we expect immunological differences to be more pronounced in the early stages and at acute onset of depression. Additionally, this approach enhances our possibilities of evaluating the CSF neuroimmunological biomarkers as prognostic biomarkers. Furthermore, the participants’ trajectories will be assessed by clinical follow-up and follow-up in the nationwide Danish registers.

### Case definition

All patients will be interviewed following the SCAN manual and in accordance to NOS we will strive for an independent diagnostic evaluation of > 95% of the patients. SCAN is a well validated and widely used instrument in assessing, measuring and classifying the psychopathology and behavior associated with the major psychiatric syndromes in adult life [40].

### Symptom rating scales

We evaluate the severity of depression by interviewer-rated scales (HAMD-17/6 and MADRS-10/6) and by the self-rating scale MDI. A detailed description of symptom rating scales is provided in eSupplementary paragraph 5.

### Risk of bias

We expect some eligible patients to decline participation solely due to the lumbar puncture. This is partly overcome by increasing knowledge of the procedure among patients and hospital staff, since lack of knowledge seems to contribute the most to unwillingness towards lumbar puncture [69].

The intervention has a time frame of approximately 3-5 h introducing the risk of the most severely depressed patients rejecting participation due to the duration of the intervention. This is overcome by breaking the inclusion down in smaller parts performed in different days and offering breaks whenever needed, and it is accepted that the most severely ill patients do not fulfill questionnaires or cognitive testing.

Furthermore, it is essential to exclude patients treated with electroshock therapy (ECT), because ECT leads to a substantial proinflammatory response and there is some evidence of induced short-term neuroinflammation [37]. Patients who receive ECT will be invited to participate three months after their last ECT session, if they still fulfill the eligibility criteria, even though this can potentially result in inclusion of patients with less severe depression.

#### Risk of recall bias

Several measures in this study are self-reported, introducing a risk of inaccuracy in data (e.g., reported weight and alcohol consumption); however, we do not expect this to differ between the groups. Additionally, we expect most of the self-reported data to be more reliable (e.g., reports of actual intake of medication) and more extensive (e.g., well-being, eating habits and exercise) than the available register data.

### Strengths and limitations

CSF is the obtainable material closest to the brain and the gold standard when conducting studies investigating immunological biomarkers of depression, and the collection of CSF samples is a substantial strength of this study. Previous studies of e.g. CSF IL-6 in depression have included far smaller sample sizes compared to this present study and the large sample size of this study is an important strength. Additionally, the thorough psychopathological evaluation and case definition by SCAN and broad symptom evaluation (discussed earlier and in eSupplementary paragraph 5) are considerable strengths due to the strong case definition and the availability of data for subgroup analyses based on symptoms. The interrater quality control of the SCAN interviews improves the quality of data. The broad variety in data collection (biological samples, thorough psychopathological evaluation, cognitive testing, level of function, quality of life and self-reported data) is also a strength as the exploratory analyses can evaluate diverse types of biomarkers for depression.

This study is to some extent limited by a potential selection bias due to willingness towards the lumbar puncture procedure (discussed previously). Furthermore, we expect the most severely ill patients to be less likely to participate (discussed previously). By including only patients with a recent onset depression and, as close to symptom debut as possible, it is aimed to minimize the proportion of included patients treated with antidepressant medication at the time of assessment. However, it is a limitation of this study that some participants are expected to be treated by antidepressants, since antidepressants have been suggested to yield immunomodulatory effects [70].

### Risks and side effects

Lumbar puncture is a commonly used procedure for diagnostics of CNS disorders and serious complications are very rare with the most common side effects being backache, nerve root irritation and headache, while infections are described only in case reports [32]. By using the atraumatic needle, the risk of inducing post lumbar puncture headache is reduced to approximately 4% [71]. We consider the procedure to be safe when carried out by a trained physician after a comprehensive neurological evaluation and screening for contra-indications. The day after the lumbar puncture, the participant will receive a phone call and any side effects will be registered. If the participant experiences severe post lumbar puncture headache, the participant will be admitted to the nearest neurological department in accordance with current practice in the Capital Region of Denmark.

### Ethical considerations

The study will be conducted in accordance with the principles of the Declaration of Helsinki (64thWMA general assembly; Fortaleza, Brazil, October 2013), and other applicable laws and regulations. Participants will always be informed of the risks listed under "Risks and side effects" in accordance to the paragraph "Information and screening assessment". It is emphasized that the participant has the right to retract the consent at any time and, for patients, that this will have no influence on future treatment in the psychiatric department/clinic. All individuals will be pseudoanonymized when analyzing the data. The identity of participants will only be used for merging of the data and retrieving biological samples. Data will only be published in a manner where no individuals can be identified.

### Unexpected findings

Lumbar puncture is routinely used for diagnosing a variety of disorders, e.g. multiple sclerosis and dementia, associated with depression [72, 73], and CSF analysis of patients initially diagnosed with depression have been reported to lead to re-diagnosis in rare cases [74]. Based on this, we expect that some of the examinations and/or samples could lead to unexpected findings regarding the health status of patients (or healthy controls). If a consent here fore has been given, the participant will be contacted and referred to further relevant investigations (e.g., a neurological department). If the findings are life threatening, the participant will be contacted regardless of consent.

### Depression in a larger perspective – the PSYCH-FLAME study

The project described in this protocol paper is part of the larger PSYCH-FLAME project investigating the immune system’s involvement in psychotic [75] and affective disorders, from a nationwide angle with large-scale register-based studies, immunogenetic studies, CSF and blood biobank studies to a thorough characterization of a cohort including 100 adults with first depressive episode and 100 adults with debut of non-affective psychotic disorders compared to at least 100 age and sex matched healthy controls. This comprehensive investigation of the associations between the immune system and mental health illnesses will hopefully pave the way for better and more precise treatment in the future.

### Perspectives and clinical implications

Large, well-conducted CSF studies are lacking and will have the potential to improve diagnostics and treatment of individuals currently diagnosed with depression, and to identify new biomarkers associated with the disorders. This study represents the largest, most comprehensive study to date investigating immunological and neurological biomarkers of patients with recent onset first time depression compared to healthy controls, and it will be the first study to investigate these biomarkers longitudinally with the potential to gain new insight into the correlations between symptoms of depression and neuroinflammation.

We expect the currently defined group of patients with depression to be divided into subgroups that are more homogenous than the current broad classification of depression. Stratification based initially on CSF biomarkers will subsequently be sought extended to blood-based biomarkers for easier clinical management. This could contribute to a paradigmatic change and pave the way for development of more effective treatments based on biomarkers in some cases, as opposed to only being based on clinical observations.

## Supplementary Information

**Additional file 1.**

## Data Availability

The data supporting findings from the study will be presented within the manuscripts and/or additional supporting files of the given publication. Person identifiable data can due to Danish legislation only be shared after approval from the Danish Data Protection Agency through a reasonable request to the study PI Professor Michael Eriksen Benros.

## Abbreviations

BACS: Brief Assessment of Cognition in Schizophrenia
BBB: Blood-brain-barrier
BDI: Beck Depression Inventory
BMI: Bodymass index
CNS: Central nervous system
CRP: C-reactive protein
CSF: Cerebrospinal fluid
ECT: Electroshock therapy
GAF: Global Assessment of Functioning
HAM-A: Hamilton Anxiety Rating Scale
HAMD-6: 6-item Hamilton Depression Rating Scale
HAMD-17: 17-item Hamilton Depression Rating Scale
ICD-10: International Classification of Diseases 10th Revision
IgG: Immunoglobulin G
IL-6/− 8: Interleukin-6/− 8
LLOQ: Lower limit of quantification
MADRS-6: 6-item Montgomery-Asberg Depression Rating Scale
MADRS-10: 10-item Montgomery-Asberg Depression Rating Scale
MDI: Major Depression Inventory
MMSE: Mini-Mental State Examination
MoCA: Montreal Cognitive Assessment
NES: Neurological Evaluation Scale
NOS: Newcastle-Ottawa quality assessment scale for case control studies
NSS: Neurological Soft Signs
PANSS: Positive and Negative Symptom Scale
PSP: Personal and Social Performance Scale
QPR: Questionnaire about the Process of Recovery
RDD: Referral- and Diagnostic Department
RCT: Randomized controlled trial
SAPS/SANS: Scale for Assessment of Positive/Negative Symptoms
SCAN: WHO Schedules for Clinical Assessment in Neuropsychiatry
SMDs: Standardized mean differences
TMT: Trail Making Test
WBC: White blood cell
WCC: White cell count
YMRS: Young Mania Rating Scale
