# Supplementary material for: Neuroimmunological investigations of cerebrospinal fluid in patients with recent onset depression – a study protocol
Source: BMC Psychiatry. 2022 Jan 12;22:35. doi: 10.1186/s12888-021-03633-0 (PMC8756720; doi:10.1186/s12888-021-03633-0)
Supplement: Supplementary file 1 — Additional file 1. [file 12888_2021_3633_MOESM1_ESM.docx]

**Neuroimmunological investigations of cerebrospinal fluid in patients with new onset depression – a study protocol**

# eSupplementary

**Contents**

**1. Recruitment**

*1.1 Outpatients*……………………………………………………………………………… 2

1.1.1 Outpatients from the Referral- and Diagnostic Department………………………….. 2

1.1.2 Outpatients from other clinics………………………………………………………… 2

1.1.3 Other outpatients……………………………………………………………………… 2

*1.2 Inpatients*………………………………………………………………………………... 3

**2. Contact to patients and obtainment of informed consent**

*2.1 Patients*………………………………………………………………………………….. 3

2.1.1 Information……………………………………………………………………………. 3

2.1.2 Screening……………………………………………………………………………… 3

*2.2 Healthy participants*…………………………………………………………………….. 4

2.2.1 Screening……………………………………………………………………………… 4

2.2.2 Information……………………………………………………………………………. 4

*2.3 Reminder*………………………………………………………………………………… 4

**3. Setting and time schedule**

*3.1 Study time schedule*……………………………………………………………………… 4

*3.2 Patient transport to the facility*………………………………………………………….. 5

**4. Biological samples**

*4.1 Blood samples*……………………………………………………………………………. 5

*4.2 Collection of cerebrospinal fluid*………………………………………………………… 6

*4.3 Procedures for biobank storage*……………………...………………………………...... 8

*4.4 Data storage and identification*……………………...………………………………....... 9

**5. Symptom rating scales**

*5.1 Hamilton depression scale - 17 items (HAMD-17)* ……………...……………………… 10

*5.2 Montgomery-Asberg Depression Rating Scale (MADRS)* ……………...……………….. 10

*5.3 Major Depression Inventory (MDI)* ……………...……………………………………… 10

*5.4 Other symptom rating scales*……………...…………………………………………........ 10

**6. Questionnaires of dietary and exercise habits**

*6.1 Dietary habits*…………………………………………………………………………………… 11

*6.2 Exercise habits*………………………………………………………………………………….. 12

7. **Simulation study on the effect of censoring**………………………………………………… 12

**8. References**……………………………………………………………………………………….. 13

**eTable 1**……………………………………………………………………………………… **5**

**1. Recruitment**

***1.1 Outpatients***

*1.1.1 Outpatients from the Referral- and Diagnostic Department*

A majority of the patients will be recruited from the Referral- and Diagnostic Department (RDD), a diagnostic clinic where patients, referred from their general practitioners suspecting a severe mental health disorder, are interviewed in order to obtain diagnosis and clarify the need for treatment. We aim for all eligible patients to be offered to participate in the study. The staff of RDD are thoroughly informed about the contents, aims and rationale of this project both in oral sessions and by posters and we aim to recap this information monthly.

*1.1.2 Outpatients from other clinics*

We also seek to include from the other outpatient clinics in the Capital Region of Denmark. The recruitment is based on the clinician informing the patient of the study. Staff in all the clinics will be thoroughly informed about the contents, aims and rationale of this project both by oral sessions and by posters. The recruitment will further be based on advertisement by flyers in the waiting rooms.

*1.1.3 Other outpatients*

If low flow of patients appears from the abovementioned strategy, patients will additionally be recruited from private psychiatrists and general practitioners. The patients will either be recruited by doctors at the departments or flyers at the facilities. Furthermore, patients will eventually be recruited from General Practice and from Depressionsforeningen (the national union of patients with depression).

***1.2 Inpatients***

Most inpatients will be recruited from the Mental Health Centre Copenhagen (MHCC) and additionally from other centers of the Capital Region of Denmark. The clinicians will initially inform the patients about the project and obtain consent to a non-binding information about the project by a research assistant.

**2. Contact to patients and obtainment of informed consent**

***2.1 Patients***

*2.1.1 Information*

Initially patients give consent to be informed about the project by one of our research assistants (all medical doctors). The patient is then given thorough oral information about the project (either by phone or in person) and if they agree to participate, a thorough written information is given as well (either physically or by email). The patient will then be given sufficient time to consider the study participation (as a minimum 24 hours), and it is emphasized that the consent can be retracted at any time.

*2.1.2 Screening*

Prior to the enrolment, the medical records are screened for eligibility. The list of diagnoses is screened for inclusion and exclusion criteria (see paragraph 2.5 “Eligibility criteria” in the main article and Table 2). Blood samples and journals are screened for contra-indications to lumbar puncture and the list of medication is screened for exclusion criteria. Language and other barriers for the SCAN interview are briefly considered as well.

***2.2 Healthy participants***

*2.2.1 Screening*

The healthy participants are screened for exclusion criteria by phone.

*2.2.2 Information*

Thorough oral information is given by phone and thorough written information is sent by e-mail.

***2.3 Reminder***

All participants will be contacted 1-2 days prior to the enrolment in order for us to remind them of the appointment and to ask if they have any questions, and to arrange the transport by taxi for the patients.

**3. Setting and time schedule**

***3.1 Study time schedule***

We aim to fit the time schedule to the patients’ needs in order to make participation as little distressful as possible, and all needed breaks are allowed. All patients will be provided lunch. An overview of the approximate time schedule is given in eTable 1. It differs from patients and healthy controls for several reasons; in order to clarify eligibility of healthy controls, the psychopathological evaluation is scheduled prior to the lumbar puncture, and we expect the initial evaluation and neurological examination to be carried out with less breaks for the healthy controls.

| **eTable 1: Time schedule.** The questionnaires will be fitted in at a suitable time. | | |
| --- | --- | --- |
| **Time a.m. (approximately)** | **Patients** | **Healthy controls** |
| 9:00 | Informed consent obtained  Initial evaluation (including evaluation of contra-indications to lumbar puncture) | Informed consent obtained  Initial evaluation (including evaluation of contra-indications to lumbar puncture) |
| 9:30 | Neurological examination | Psychopathological evaluation |
| 10:00 | Blood samples | Neurological examination |
| 10:30 | Lumbar puncture | Blood samples |
| 11:00 | Lunch break | Lumbar puncture |
| 11:30 | Psychopathological evaluation | Cognitive testing |
| 13:00 | Cognitive testing |  |
| At home* | Fecal sample | Fecal sample |
| *The fecal sample can be collected at the CORE facility if the participant prefers so. | | |

***3.2 Patient transport to the facility***

All patients will be offered transport by taxa forth and back to the facility to minimize discomfort from participation.

**4. Biological samples**

***4.1 Blood samples***

1. The patient is seated in a comfortable way (laid down if preferred)
2. A tourniquet is added to the arm
3. The skin area is cleaned by an 82% ethanol/0,5% chlorhexidine swap (Alkoholswabs, Mediq Danmark, Brøndbyvester, Denmark)
4. Using a sterile culture swab (BD BBL CultureSwab, BD BBL, Brescia Italy) a swab sample will be taken of the skin above the point where the blood sample will be taken
5. A SAFETY Blood Collection Set + Holder 21G x ¾’’ (Greiner Bio-One Gmbh, Kreimsmünster, Austria) needle appropriate for collecting venous blood is used to puncture one of the venes in fossa cubiti
6. The following samples are taken in the following glasses (Vacuette® Tube, Kremsmünster, Austria):
   1. 1 x 4 ml Lithium Heparin Sep
   2. 1 x 2 ml FX Sodium Fluroide/Potassium Oxalata
   3. 2 x 2 ml K2E K2EDTA
   4. 3 x 6 ml K3E K3EDTA
   5. 3 x 6 ml Z serum Clot Activator
7. All glasses will be labeled immediately after collection for later identification (PSYCH-FLAME identification ID, name, CRN)
8. The tourniquet is removed, and the needle is retracted
9. Any bleeding is stopped with cotton wool
10. Blood samples are stored at room temperature until CSF samples are available and transported together to Department of Clinical Biochemistry, Rigshospitalet, University Hospital of Copenhagen

***4.2 Collection of cerebrospinal fluid***

After it has been clarified by examination (questions and neurological examination) that there are no contra-indications to lumbar puncture, and the blood samples have been collected, lumbar puncture will be carried out according to current consensus. It will be noted in the database if the participant has taken anxiolytics prior to lumbar puncture.

1. The procedure will be explained in detail to the participant, including the risks following lumbar puncture (post lumbar headache, infection and bleeding)
2. The patient is put in lateral decubitus position with approximately 100-degree flexed hip. It is made sure that the patient is comfortable. Parallelism of the anterior superior cristae iliacae spines, knees, and shoulders are sought. If it appears difficult to perform the lumbar puncture in this position, the patient will be in a seated position instead. The position will be noted in the database.
3. The lower back is inspected and the L4 spinous process is identified by measuring from the upper part of the hips.
4. The examiner will put on sterile gloves (ProFeel® DHD™ Micro Powder Free Latex Surgical Gloves, WRP, Vienna, Austria)
5. In the meantime, the assistant prepares a sterile area on a suitable table with a Barrier® sterile cover (Barrier, Göteborg, Sweden). On this, sterile items for the further procedure are placed (see below)
6. The skin area covering the space between the L3 and L4 or L4 and L5 spinous processes is cleaned by using 0.5% chlorhexidin-digluconat/83% v/v ethanol in cleaned water (Region Hovedstadens Apotek, Herlev, Denmark). The liquid is applied by using a sterile Dressing set (Bastos Viegas, Gullhufe, Portugal) starting over the L3/L4 or L4/L5 space moving outwards in a spiral. This is repeated once
7. Using a sterile culture swab (BD BBL CultureSwab, BD BBL, Brescia Italy), a swab sample will be taken of the skin above the point where the lumbar puncture will be performed
8. A Barrier® sterile cover (Barrier, Göteborg, Sweden) with a hole in the center is attached to the patient leaving a sterile area for lumbar puncture
9. The examiner will now re-identify the L3-L4/L4-L5 space and local anesthesia will be given with lidocaine (2-5 ml depending on the weight of the participant) (Lidokain Mylan 20 mg/ml, Mylan Hospital AS, Oslo, Norway) retracted from the container with a 23G cannula (KDM® KD-FINE®, Berlin, Germany) attached to a 5 ml syringe in a sterile procedure
10. While maintaining sterility, the lidocaine will be injected in the subcutis covering the area over the L3-L4/L4-L5
11. After waiting 5 minutes for the lidocaine to work, the lumbar puncture will be carried out using an atraumatic 22G needle (RapID™ Spinal Needle Set Pencil Point Spinal Needle, Smiths Medical International Ltd., CT21, 6JL, UK)
12. Collection of CSF will be started immediately. One ml CSF is estimated to be 20 droplets
    1. The first ml of CSF will be discarded, in order to minimize the amount of blood in the samples
    2. The next two ml of CSF is collected one ml at a time in two 5 ml tubes (Sarstedt, Nümbrecht, Germany)
    3. The following 14 ml of CSF will be collected in a suitable 15 ml non-pyrogenic tube (Sarstedt, Nümbrecht, Germany)
13. All glasses will be labeled immediately after collection for later identification (PSYCH-FLAME identification ID, name, CRN)
14. As soon as the CSF samples are available, they will be driven by taxa along with the matching blood samples and be analyzed within a maximum of 1 hour from lumbar puncture (preferable half an hour) and samples are kept at room temperature and in a zip-bag
15. The time from lumbar puncture to analysis is noted in the database
16. Meanwhile the needle is retracted and placed in a suitable safe container
17. The skin is covered with an adhesive surgical dressing (evercare®, OneMed, Helsinki, Finland)

The participant will be offered something to drink before proceeding and, even though the evidence is sparse, be encouraged to drink caffeine drinks and water for the next 24 hours, around 2 liters in total. We will advise the patient to refrain from hard physical activity the next 12 hours and to avoid public swimming pools within the next couple of days (until the wound is visually heeled). No other restrictions will be given.

***4.3 Procedures for biobank storage***

1. The following procedures will be carried out within an hour from the lumbar puncture. A total of 36 ml blood and 14 ml CSF will be handled in accordance to the following:
2. Time of sample arrival/procedure start is noted
3. 6 PKU cards (filter papers, Schleicher & Schuell, Dassel, Germany) are marked with PSYCH-FLAME ID and date
4. 100 µL from the top of the two of the three Z serum clot activator tubes are pipetted and placed on the PKU card marked “Serum før G”
5. 100 µL from the top of the two of the three K3EDTA tubes are pipetted and placed on the PKU card marked “EDTA før G”
6. 2 x 100 µL from the top of the Sarstedt tube containing CSF are pipetted and placed on the PKU card marked “CSF før G”
7. Samples are centrifuged at 22ºC at 3200 rpm (1145 G) for 10 minutes with standard acceleration and deceleration (Universal 320R, Hetteich Zentrifugen)
8. The periphery of the semi-dry CSF spots is marked
9. Pre-printed labels are put on Nunc Cryo vials (Greiner Bio-one) (11 with PSYCH-FLAME ID and “serum”, 13 with PSYCH-FLAME ID and “EDTA” and 28 with PSYCH-FLAME ID and “CSF”)
10. The storage box is marked with PSYCH-FLAME ID and date by preprinted labels
11. When the centrifugation has ended, the samples are carefully removed from the centrifuge
12. 100 µL supernatant from two of the three Z serum clot activator tube are pipetted and placed at the PKU card marked “Serum efter G”
13. 100 µL supernatant from two of the three K3EDTA tubes are pipetted and placed at the PKU card marked “EDTA efter G”
14. 2 x 100 µL from the supernatant of the CSF sample are pipetted and placed on the PKU card marked “CSF efter G”
15. The PKU cards are kept at room temperature overnight
16. 500 µL aliquots from the supernatants of the three Z serum clot activator tubes are transferred to the Nunc Cryo Vial marked “serum” and repeated until there is less than 500 µl supernatant left (usually 11 cryo vials)
17. 500 µL aliquots from the supernatants of the three K3 EDTA tubes are transferred to the Nunc Cryo Vial marked “EDTA” and repeated until there is less than 500 µl supernatant left (usually 13 cryo vials)
18. 500 µL aliquots from the top of the CSF sample are added to the cryo vials marked with PSYCH-FLAME ID and “csf” (usually 28 cryo vials)
19. The tubes are stored in the box
20. The periphery of the semi-dry CSF spots on the second CSF PKU card are marked with a pen
21. All samples (except the PKU cards that are placed in the lab to dry at room temperature overnight but including the pellet of the 3 Z serum clot activator tubes and 3 K3EDTA tubes) are moved in the storage box and stored directly at -80ºC
22. The next morning the dry PKU cards are stored in separate, small plastic bags and then placed in the storage box of this specific participant at -80ºC

Until participant 70 all preparation for biobank storage is carried out in Statens Serum Institut. All later samples are prepared for biobank storage at Department of Clinical Biochemistry, Rigshospitalet.

***4.4 Data storage and identification***

The social security number, a unique Civil Registration Number (CRN) which is assigned to all Danish citizens at birth or at the time of immigration, is used to identify all patients when contacted by a research assistant from the PSYCH-FLAME study. Patients who give informed consent will be registered by their CRN and pseudoanonymized by an identification key kept elsewhere, as according to the approval from the Danish Data Protection Agency. This will make it possible to later expand the project with data from the Danish registers.

**5. Symptom rating scales**

***5.1 Hamilton depression scale - 17 items (HAMD-17)***

HAMD-17 is a well-established depression scale established by Hamilton in 1967 and is intended for in-patients with unipolar depression (1). The HAMD-17 scale has been widely used for research purposes (e.g. studies evaluating inflammatory markers in CSF (2–4)) and is used on daily basis by clinicians. The widespread use of this rating scale enhances interpretation and reevaluation of our results.

***5.2 Montgomery-Asberg Depression Rating Scale (MADRS)***

MADRS is a sub-scale consisting of 10 items concerning depressive symptoms derived from the Comprehensive Psychopathological Rating Scale (5). It is not as commonly used in studies of depression as HAMD-17 but is however characterized by less focus on physical symptoms and is added here to supply the information from HAMD-17.

***5.******3 Major Depression Inventory (MDI)***

MDI is a 10 items patient-administered questionnaire developed by a Danish WHO Collaborating Centre and covers both ICD-10 and Diagnostic and Statistical Manual of Mental Disorders (6). Several other self-rating scales are available including the most widely used Beck Depression Inventory (BDI). However, MDI has been shown to be superior to BDI with a higher homogeneity (7).

***5.4 Other symptom rating scales***

Anxiety is a common co-morbid condition to depression (8) and symptoms of anxiety will be rated separately on the HAM-A 14 items scale. To evaluate patients with psychotic depression, symptoms will be rated on SAPS, SANS and PANSS. Patients will also be rated on YMRS to evaluate manic symptoms.

**6. Questionnaires of dietary and exercise habits**

All recorded items regarding dietary habits and exercise are self-reported, and the questions are described below.

***6.1 Dietary habits***

Dietary habits are reported on the following scale:

Never – 1-3 per week – 4-6 per week – 1-2 per day - >2 a day – unknown

This scale applies to the following items:

1. Fats on bread: butter, margarine, fat, no fats on bread.
2. Toppings: cold cuts/meat, fish, eggs, mayonnaise-based salads.
3. Hot meat: meat, poultry, fish.
4. Vegetables: cooked vegetables, mixed salads, other raw vegetables.
5. Fats for cooking: margarine, butter, fat, olive oil, corn/sunflower/grape seed based oils, rape seed/other oils, without fat.

Portions of fruits are evaluated on the following scale:

Never – 1-2 per week – 3-4 per week – 5-6 per week – 1-2 per day – 3-4 per day – 5-6 per day - >6 per day – unknown.

***6.2 Exercise habits***

The participants are asked about the following

1. Moderate and hard exercise, hours per week.
2. Hard exercise, hours per week.
3. Sitting time, transport, hours per day.
4. Sitting time, work/school, hours per day.
5. Sitting time, screen, hours per day.
6. Sitting time, other free time, hours per day.

**7. Simulation study on the effect of censoring**

An effect-size (SMD) of 0.40 gives 80% power in a two-sample t-test with alpha=5% and n=100 in each group.

We simulated 100 samples from X1 ~ N(0, 1) and 100 samples from X2 ~ N(0.4, 1) and confirmed that a two-sample t-test is significant at the 5% level in 80% of the simulations.

Censoring observations below a lower limit of quantification (LLOQ) such that on average 33% are censored: P(X1 < LLOQ)/2 + P(X2 < LLOQ)/2 = 33% (thus LLOQ = -0.250) and analyzing the censored observed observations using the censored Gaussian model the test of a group difference was significant in at least 77% of cases thus corresponding to a (80-77)/80*100% < 5% reduction of power.

Censoring observations below a lower limit of quantification (LLOQ) such that on average 50% are censored: P(X1 < LLOQ)/2 + P(X2 < LLOQ)/2 = 50% (thus LLOQ = 0.20) and analyzing the censored observed observations using the censored Gaussian model the test of a group difference was significant at least 72% of cases thus corresponding to a (80-72)/80*100% = 10% reduction of power.

In all cases we used 10,000 simulations.

**8. References**

1. HAMILTON M. A rating scale for depression. J Neurol Neurosurg Psychiatry. 1960 Feb;23(1):56–62.

2. Sasayama D, Hattori K, Wakabayashi C, Teraishi T, Hori H, Ota M, et al. Increased cerebrospinal fluid interleukin-6 levels in patients with schizophrenia and those with major depressive disorder. J Psychiatr Res. 2013 Mar;47(3):401–6.

3. Palhagen S, Qi H, Martensson B, Walinder J, Granerus A-K, Svenningsson P. Monoamines, BDNF, IL-6 and corticosterone in CSF in patients with Parkinson’s disease and major depression. J Neurol. 2010 Apr;257(4):524–32.

4. Ishii T, Hattori K, Miyakawa T, Watanabe K, Hidese S, Sasayama D, et al. Increased cerebrospinal fluid complement C5 levels in major depressive disorder and schizophrenia. Biochem Biophys Res Commun. 2018 Mar;497(2):683–8.

5. Montgomery SA, Asberg M. A new depression scale designed to be sensitive to change. Br J Psychiatry. 1979 Apr;134:382–9.

6. Olsen LR, Jensen D V, Noerholm V, Martiny K, Bech P. The internal and external validity of the Major Depression Inventory in measuring severity of depressive states. Psychol Med. 2003 Feb;33(2):351–6.

7. Konstantinidis A, Martiny K, Bech P, Kasper S. A comparison of the Major Depression Inventory (MDI) and the Beck Depression Inventory (BDI) in severely depressed patients. Int J Psychiatry Clin Pract. 2011 Mar;15(1):56–61.

8. Jacobi F, Wittchen H-U, Holting C, Höfler M, Pfister H, Müller N, et al. Prevalence, co-morbidity and correlates of mental disorders in the general population: results from the German Health Interview and Examination Survey (GHS). Psychol Med. 2004 May;34(4):597–611.
